# Harassment disparities and risk profile within lesbian, gay, bisexual and transgender Spanish adult population: Comparisons by age, gender identity, sexual orientation, and perpetration context

**DOI:** 10.3389/fpubh.2022.1045714

**Published:** 2022-12-15

**Authors:** José Devís-Devís, Sofía Pereira-García, Alexandra Valencia-Peris, Anna Vilanova, Javier Gil-Quintana

**Affiliations:** ^1^AFES Research Group, Departament d'Educació Física i Esportiva, Universitat de València, València, Spain; ^2^AFES Research Group, Departament de Didàctica de l'Educació Física, Artística i Música, Universitat de València, València, Spain; ^3^GISEAFE Research Group, Institut Nacional d'Educació Física de Catalunya (INEFC), Universitat de Barcelona, Barcelona, Spain

**Keywords:** gender identities, sexual minorities, harassment, discrimination, disadvantage

## Abstract

Lesbian, Gay, Bisexual and Transgender (LGBT) harassment disparities have become a public health issue due to discrimination and the effects on these people's health and wellbeing. The purpose was to compare harassment disparities within the Spanish adult LGBT population according to age, gender identity, sexual orientation and the context of perpetration and to describe the harassment risk profile. A sample of 1,051 LGBT adults participated in a cross-sectional study. Frequencies, percentages and Chi-square tests of independence for stablishing significant differences (*p* < 0.05) were calculated. The corrected standardized residuals allowed to identify the categories in which significant differences emerged. Binomial logistic regression was used to define the probability of the main LGBT groups of suffering harassment. Results show that 54.4% of the participants had experienced harassment. Young adults presented a higher prevalence than the older group. There were significant harassment differences between transgender (67.2%) and cisgender (52.7%) groups, and also between the subgroup of trans women (75.8%) and the subgroups of cis men (60.2%) and cis women (42.9%). The main disparities according to sexual orientation emerged between lesbian trans and the other LGB groups. Most harassment occurred in educational contexts and public spaces. Trans-women and trans non-binary reported a higher rate of harassment than cis LGB persons in all contexts. Trans people with different orientations (especially lesbian and gay trans) differed in harassment from LGB cis in four of the six contexts analyzed. Harassment is likely to diminish between 2 and 3% each year as LGBTs get older in educational contexts and public spaces but increases 1.07 times in the workplace. Trans women, trans non-binary, lesbian cis and trans-men were more likely to suffer harassment than bisexual cis persons. Trans women present the highest risk of harassment in three contexts (workplace, family and public spaces) and trans non-binary in the other three contexts (education, health and sport). Harassment is a serious problem for LGBT adults in Spain, especially among trans people, which differ in characteristics from those of the sexual minorities mainstream. Programs and policies targeted for improving health should therefore consider the differences that came to light in this study.

## Introduction

Lesbian, gay, bisexual and transgender (LGBT) persons shape a community of people that feel discriminated by their sexual orientations and/or gender identities. Lesbians and gay persons are emotionally and sexually attracted by same sex persons, women the former and men the latter, while bisexuals are attracted, in the same way or degree, by more than one sex and not necessary simultaneously. These three groups of persons are cisgender when their gender identities are aligned with their sex at birth. Trans people are a varied group of persons with diverse gender identities and/or expressions that differ from those cultural expectations linked to the sex assigned at their births, and have different sexual orientations such as heterosexual, bisexual, homosexual (gay or lesbian) and others (1). All LGBT people are an under-represented and underserved population in many research fields such as health disparities despite this type of research is continually increasing (2, 3). Disparities are not only about differences among groups of people, but also about the special type of differences that adversely affect disadvantaged groups (4). According to Braveman (5), the concerns at the heart of this concept are about social justice with respect to the treatment of privileged as compared to disadvantaged people. This concept has been used in relation to medical health worries since the end of the Twentieth Century (6, 7) and more recently has been applied to social health disparities such as harassment issues, particularly to LGBT harassment (8, 9). In fact, research interest in the harassment suffered by LGBT people due to their sexual or gender identities has increased in recent years (10–12).

According to some studies, harassment and discrimination are ongoing and prevalent worldwide among the LGBT community (13). In Europe, for instance, an international study indicates that 38% of LGBT respondents experienced harassment in the last 12 months and 58% over the last 5 years, with similar data from Spain (41 and 57%, respectively) (14, 15). Discrimination and harassment of these people in the USA is also significant, since 57% of them have faced slurs and 53% received offensive comments because of their gender identities or sexual orientations (16). In Australia, 85% of this community has experienced harassment, violence or homophobic abuse at some time in their lives (17). Latin America has one of the highest rates of violence against this population, even after the creation of laws designed to protect LGBT people against discrimination in many countries (18, 19). In the Middle East, North Africa and Central and Southern Asia, the situation of LGBT people is even worse because homosexuality is either forbidden or socially unacceptable and many of them are targets of violence and discrimination (20, 21). A recent worldwide report indicates that these persons still suffer arrest and prosecution for consensual same-sex sexual acts or for diverse gender expressions in at least 29 United Nations Member States (22).

Apart from its global prevalence, harassment disparities experienced by LGBT people differ within heteronormative societies by age, gender identity, sexual orientation and perpetration contexts (e.g., education, family, work, health, sport and public spaces) (10, 14, 23).

## Harassment disparities and risk of harassment

The prevalence of harassment by age among European LGBT people is 47% among those between 15 and 17 years old, similar in Spanish youth (49%), while the whole community presents 9 percentage points less harassment (14, 15). Younger USA generations of LGBT people also experience more harassment and discrimination than older ones, with a higher percentage among Generation Z (under 26 years old) (57%), followed by Generation Y (between 26 and 41 years old) (42%), Generation X (between 42 and 57 years old) (30%) and Baby Boomers (between 58 and 76 years old) who are members of the generation with the lowest percentage (20%) (10). Among Spanish trans people this difference is according to the self-defined gender disclosure age instead of the chronological age. Those who disclose their gender at a younger age, under 16 years old, suffer more harassment than those who do so later (24). Predictive data indicate that young LGBT people are more likely to experience harassment and discrimination than older members of this community (23, 25).

Harassment disparities are also founded by gender identities since transgender people experience more harassment and discrimination than cisgender people. For instance, trans people from Europe and the USA show higher percentages (48 and 62%, respectively) than the whole LGBT community (38 and 36%, respectively) (10, 14). This difference is even observed between transgender or trans persons (80%) and their non-trans siblings (63%) (26). More women suffer harassment and discrimination than men, even if they identify themselves as trans (24, 26) or cisgender persons (8, 25).

According to sexual orientation, more LGBT youth and adults experience and are more likely to experience harassment and violence than their non-LGBT peers (25, 27, 28). Within this community, more young lesbian/queer girls (72%) experience harassments than bisexuals (66%) and gay/queer boys (66%), although trans people of any sexual orientation have the highest prevalence of harassment (81%) (29). In general, more LGBT people of different ages experience harassment and victimization than heterosexual people (25, 27, 28).

Educational settings (school, higher school or university) attract a considerable amount of research on LGBT harassment. Several empirical studies in the USA and abroad suggest that young people of this community have significantly higher rates than their heterosexual and cisgender peers in educational contexts (27, 30–34). Mahowald et al. (10) found the global prevalence in US schools ranges from 21% of harassment or discrimination to 68.7% of verbal and 34.2% of physical harassment found in Kosciw et al. (35). Whitfield et al. (23) reported 44.9% of harassment in schools, 57.5% being bisexuals, 56.7% queers, 43.1% gays and 42.3% lesbians (no transgender in the sample). This prevalence is not too far from the 51% of harassment found in European schools (14) and also close to 49% in Spanish schools on a daily and frequent base (36). Many LGBT people are harassed by their classmates (86.3%), especially in spaces unmonitored by teachers (33, 35). These incidents increase when LGBT students display other marginalized identities such as a minority ethnicity or religion (30). In the particular case of trans people, the prevalence among K-12 students in the USA is 77% in verbal harassment, and physical and sexual assaults (37), while the prevalence of harassment in these persons in Spanish educational contexts is 46.2% (24). As in the whole LGBT community, some studies indicate that trans youths also suffer more harassment than their LGB peers in schools (38, 39).

Families are also involved in harassment, especially as regards young LGBT people, either helping to overcome it or engaging in it. The studies by D'Augelli et al. (40) and Katz-Wise et al. (28) suggest that family acceptance of LGBT members in the US is essential for their wellbeing and health since it reduces the internalized homophobia and can prevent the need for mental health care. However, prevalence of harassment experienced by LGBQ at home is 83.4% of US respondents in the study by Whitfield et al. (23), those over 24 years old being between 56 and 90% less likely to experience harassment. 57% of transgender people from this country, in particular, experience significant family rejection (41) and 31.1% of trans persons in Spain suffered harassment in their family settings (24). According to Juárez-Chávez et al. (42), violence and mistreatment of transwomen and gay men from family members in Peru is perpetrated by having feminine preferences during their childhood. In these cases, “gender stereotypes and gender non-conformity are key determinants of the violence experienced” (p. 6).

Harassment and bullying are also rooted in the organizational culture of the workplace. According to Hollis and McCalla (43) this problem can potentially affect 7 million LGBT workers in the USA. In fact, 36% of these people in the US experience harassment or discrimination (10) and 37% of lesbians and gays have experienced harassment in their workplace in the last 5 years and 12% lost a job because of their homosexuality (44). According to Sears at al. (11), 37.7% of LGBT people experience at least one form of harassment in the US, transgender being significantly more likely to report verbal harassment than cisgender LGB employees (43.8% compared to 29.3%). 15% of trans people are verbally harassed and physically or sexually assaulted in the US (37), while the percentage is 21.7% in different Spanish labor contexts at some time in their working lives (24).

LGBT people also experience harassment, micro-aggressions and stigma in health contexts, including inappropriate curiosity by healthcare personnel (45, 46). They are also afraid of being discriminated against because of their sexual orientation or gender identity and 18% do not seek medical care when they need it to avoid homophobia and transphobia (16). Transgender people in the US in particular report 33% negative experiences, including verbal harassment, refusal of treatment and physical and sexual assaults (37), while 24.1% of these persons experience harassment in the Spanish health system (24).

According to an international study with non-heterosexual participants from six English-speaking countries, 54% of gay men and 48% of lesbian women have suffered homophobia in sports environments (47). In Europe, 90% of LGBT people still perceive that homophobia and transphobia are a current problem in sport (48). In a recent European study, 81.9% of them faced verbal insults, 36.2% experienced unacceptable physical conduct and 20.1% physical violence in sport (49). In a study carried out on Spanish trans persons, 18.87% of the participants experienced harassment in sports contexts (24).

Some studies point out that public places are contexts in which most LGBT people suffer harassment and they are more likely to report it than their heterosexual and cisgender peers (14, 23, 50). The prevalence of harassment is 51% in public spaces in the US (10), reaching 93.7% in the streets and particularly affecting 95.4% of gays, 95.1% queers, 93.8% lesbians and 91.4% bisexuals (23). In Europe, 42% of LGBT respondents in a survey (14) experienced harassment. Particularly, 31% of trans people experience at least one type of mistreatment in public accommodation in the US (37), and 49% of trans participants in a Spanish study, especially trans women (55,9%), suffered harassment in these contexts (24). This sort of harassment is usually committed by strangers and consists mostly of micro-aggression, disapproving looks and comments that they frequently receive walking down the street or holding hands with a partner in public (14, 51).

Harassment environments lead to many negative consequences for LGBT people that hinder their personal development and their careers. In a heteronormative society, being socially perceived as deviant from the traditional social roles of masculinity and femininity and gender stereotypes is cause for rejection and punishment (14, 23). Skipping school, poor academic performance, worse health, symptoms of depression and anxiety were some of the negative consequences of harassment at school (30, 34, 41, 52). In family contexts, a negative parental response also contributes to depression, while extreme family conflicts can lead to some LGBT youths becoming homeless (53, 54). In the workplace harassment and bullying can produce disengagement at work, unfocused attention and time off sick with the consequent considerable cost for the organization. And since sports and health contexts are not perceived as safe spaces, some LGBT people withdraw from them, worsening their wellness and health (45, 47).

Against this backdrop, the main purpose of this paper is to compare harassment disparities within the Spanish adult LGBT population, according to age, gender identity, sexual orientation and perpetration context. This extends the disparities perspective beyond a simple comparison with the heterosexual population and focuses on differences among the groups in the whole adult LGBT community. It is especially relevant because we introduce the “T” to the comparison, since this collective is largely invisible in many studies. Although harassment has been analyzed among these minorities in Spain according to the context of perpetration, it has not included gender identity and sexual orientation within this community. We specially focused on adults instead of adolescents (55), although a few exceptions have been identified exclusively on youth and adult trans people (24). The paper's secondary purpose is to describe the risk profile of harassment in LGBT people, based on the probability of experiencing this behavior, to compare the groups in this community (lesbian, gay, bisexual and transgender) by age and the perpetration context.

## Methods

### Participants

An initial sample of 1,658 participants aged 18 to 74 from different regions of Spain participated in a wide project designed to assess experiences of different psychosocial issues in LGBT persons. Data were collected during 2019 and early 2020. Only participants who defined themselves as trans people or as LGB cisgender persons were included in this cross-sectional study. Six hundred and seven participants were excluded because they had not answered the questions related to harassment experiences (*n* = 489) and because they were non-LGB cisgender persons (*n* = 118), corresponding to 1051 participants the final sample.

For the comparative purpose of this paper, three subgroups of gender identities for transgender people (T) (trans men, trans women and trans non-binary) and three subgroups for cisgender participants (LGB) (cis men, cis women and cis non-conforming) were identified. Following Human Rights glossary of terms (https://www.hrc.org/resources/glossary-of-terms), in this paper non-binary refers to those trans people who identify themselves with a gender which is in-between, fluctuating or beyond the two categories “(trans)man” and “(trans)woman”, or completely outside these categories (as having no gender, bi gender or gender fluid). The non-conforming term refers to those cis people who behave differently to the traditional ways of their gender, or whose gender expression does not fit clearly into a category. Moreover, five sexual orientation categories were identified for transgender people (heterosexual, lesbian, gay, bisexual and other) and four for cisgender participants (lesbian, gay, bisexual and other).

### Measures and procedure

A digital questionnaire was compiled on LimeSurvey (Version 2.73.1+) to obtain information about different psychosocial issues related to LGBT people's experiences at different contexts of their daily life. The scales included were validated in Spanish language in previous studies, but for the purposes of this paper only data for perceived harassment items were used. As previously described (24), the information on harassment and contexts of harassment (workplace, educational, health, sports, family setting and public spaces) was included in the survey. In particular, LimeSurvey presents a question on the harassment experienced (“Have you ever experienced any kind of harassment based on gender and/or sexual orientation or identity?—Harassment refers to aggressions received from other people ranging from annoyance to serious abuse that may be intimidating and/or violate personal dignity”) and participants answer with a YES or NO. When they respond affirmatively, an open window appears with table responses referring to the six contexts of harassment.

The sample was mostly accessed through ~200 Spanish activist LGBT associations, which are committed to avoiding discrimination, promoting visibility and protecting LGBT rights in areas such as health, workplace, education, family and/or sports. An email explaining the purpose of the study and containing a link to the questionnaire was sent to these associations, which in turn redistributed it among their members and workers. This strategy has been used in other studies with hidden or low visibility groups such as sex workers, drug consumers or trans people (56–58). The completely anonymous and voluntary questionnaire was also spread by posting recruitment advertisements in social media (e.g., Twitter and Facebook). Materials and procedures were approved by the Ethics Committee of the the University of Valencia and the Catalan Sports Council, as part of a joint project between research groups from different institutions, to guarantee ethical principles in social research on human beings. Informed consent form authorizing the research team to publish the data collected was approved online by the participants before accessing the questionnaire.

### Data analysis

Variables included were (a) harassment (experienced or not); (b) harassment contexts (workplace, educational, health, sport, family, and public spaces); (c) main gender identity groups (cisgender and transgender); (d) particular gender identity groups for trans persons (trans women, trans men or trans non-binary); (e) particular gender identity groups for cis people in this sample (cis women, cis men or cis non-conforming); (f) sexual orientation for transgender people (heterosexual trans, gay trans, lesbian trans, bisexual trans and other trans); (g) sexual orientation for cisgender persons (lesbian cis, gay cis, bisexual cis and other cis); and (h) age (continuous and categorical: four groups).

The data were analyzed on IBM SPSS 26.0 statistical software. Variables were encoded and the data were typed into the program. The statistical analysis consisted of the calculation of frequencies and percentages and the findings were presented in figures and tables. Chi-square tests of independence were carried out to reveal the existence of significant differences (*p* < 0.05) among the variables. The corrected standardized residuals were calculated to identify the categories with significant differences (corrected standardized residuals ±1.96). To determine the effect size of the Chi-square analyses, Cramer's V coefficient was used as a measure of the strength of the association, where ≥0.1, ≥0.3, and ≥0.5 represent a weak, moderate, or strong association, respectively. Binomial logistic regression was used to define the probability of experiencing harassment by the main groups of gender (cisgender and transgender), and sexual orientation, age and contexts. The odds ratios were also determined using 95% confidence intervals (CI).

## Results

### Descriptive characteristics of the sample

The characteristics of the final sample (*n* = 1051) can be seen in Table 1. Most participants were distributed quite similarly in the three young range ages while only few participants were over 50 years old. The sample was predominantly composed of cisgender people with 12% of transgender persons. Most of cisgender participants were men, followed by a third of them who were women, and only a few were gender non-conforming persons. On the contrary, transgender were likewise distributed among trans women, trans men and trans non-binary, although men showed the highest percentage of participants. Moreover, transgender people were distributed quite similarly among the five sexual orientations identified. Regarding cisgender participants, gay cis predominated over lesbian and bisexual cis, corresponding the low percentage of participants to those identified with “other” orientations.

**Table 1:** Characteristics of the LGBT sample.

	***n* (%)**
**All sample**	1,051 (100.0)
**Age groups**	
18–24	288 (27.4)
25–34	333 (32.1)
35–50	331 (31.9)
≥ 51	86 (8.3)
**Gender**	
**Transgender**	128 (12.2)
Trans women	33 (3.1)
Trans men	58 (5.5)
Non-binary	37 (3.5)
**Cisgender**	923 (87.8)
Woman	375 (35.7)
Man	525 (50.0)
Non-conforming	23 (2.2)
**Sexual orientation**	
**Transgender**	128 (12.2)
Heterosexual	23 (2.2)
Gay	13 (1.2)
Lesbian	15 (1.4)
Bisexual	38 (3.6)
Other	39 (3.7)
**Cisgender**	923 (87.8)
Gay	473 (45.0)
Lesbian	216 (20.6)
Bisexual	184 (17.5)
Other	50 (4.8)

### Harassment by age, gender identity, and sexual orientation

The results showed that 54.4% of the LGBT participants had experienced harassment at some time in their lives. The percentages of harassment varied between the age groups (χ^2^ = 11.528, *p* < 0.01, *Cramer's V* = 0.105). The study of corrected standardized residuals revealed that more 25–34 years-old participants had suffered harassment (61.3%) than the older group >50 years (44.2%). Harassment was also influenced by the main gender groups within the Spanish LGBT community, since Chi-square tests revealed differences between the transgender (67.2%) and cisgender (52.7%) groups (χ^2^ = 9.572, *p* < 0.01, *Cramer's V* = 0.095).

**Figure 1:**
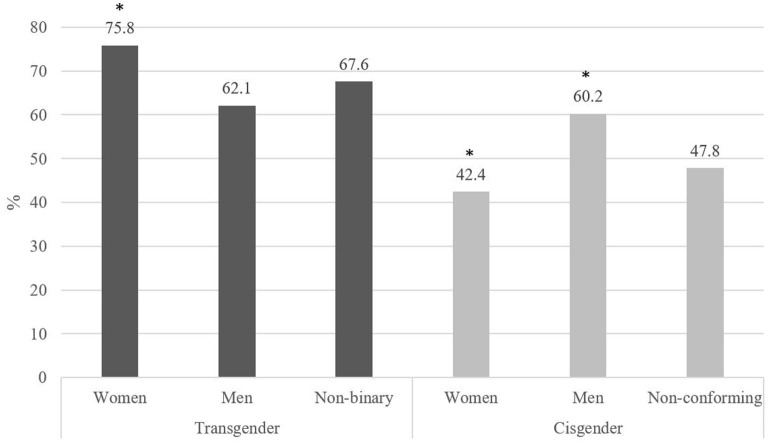
Percentage of harassment by gender identity.

The analysis of harassment disparities by particular gender groups (trans women, trans men, trans non-binary, cis women, cis men and cis non-conforming) is shown in Figure 1. The Chi-square test revealed differences by gender group (χ^2^ = 39.298, *p* < 0.001, *Cramer's V* = 0.193). The study of corrected standardized residuals showed that transgender women, the group with the highest rate of harassment (3 out of 4 had suffered it), differed significantly from cisgender women, the group with the lowest rate of harassment (42.4%), and cisgender men (60.2%).

**Figure 2:**
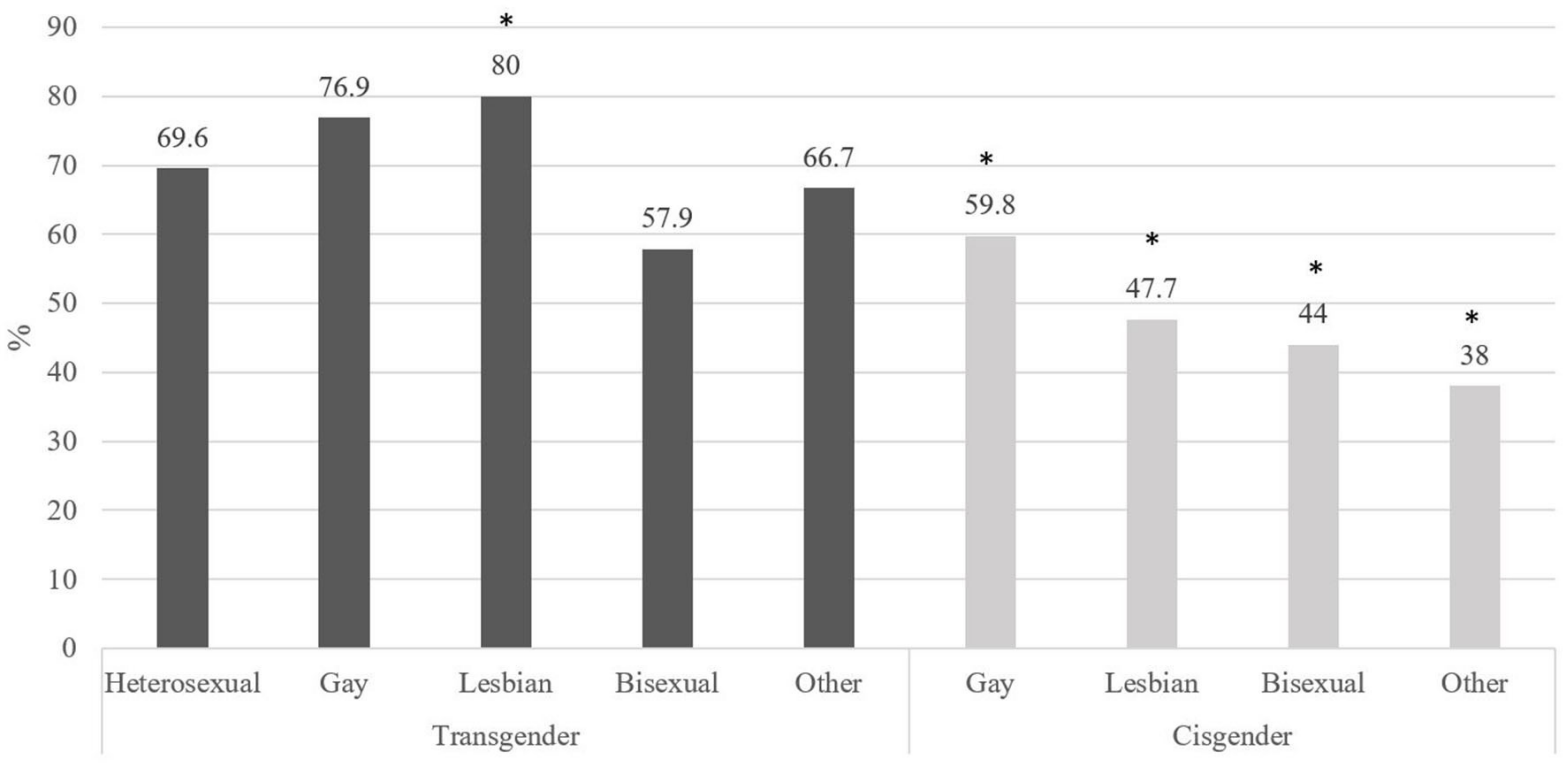
Percentage of harassment by sexual orientation.

The analysis of harassment by sexual orientation groups (transgender people divided into heterosexual, gay, lesbian, bisexual and other; and cisgender persons in gay, lesbian, bisexual and other) is presented in Figure 2. The Chi-square test revealed differences between the nine groups (χ^2^ = 34.270, *p* < 0.001, *Cramer's V*=0.181). The study of corrected standardized residuals showed significant differences between the lesbian transgender (the group with the highest rate of harassment) and all the cisgender groups (gays, lesbian, bisexual and other sexual orientations), who reported lower levels of harassment.

### Harassment by contexts

LGBT people have suffered harassment in different contexts and social areas, as shown in Table 2. Most of the harassment experienced by the participants in this study was in the educational context (school, high school or university) with 45%, as against 43.5% in public spaces. They are followed by another group of contexts with similar percentages of harassment, such as the workplace (21.2%), sports (19.6%) and the family (17.9%). The health context was the one in which the smallest percentage of harassment was experienced by the participants, with 10.9% of the total sample. 18% of the participants (*n* = 102) suffered harassment in only one context, 50.2% (*n* = 285) in two or more contexts, and 31.9% (*n* = 181) suffered it in four or more contexts.

**Table 2:** Harassment contexts by gender identity and sexual orientation groups.

	**Workplace**	**Educational**	**Health**	**Sports context**	**Family**	**Public spaces**
**All sample**	223 (21.2)	473 (45)	115 (10.9)	206 (19.6)	188 (17.9)	457 (43.5)
Trans	**43 (33.6)**	**72 (56.3)**	**45 (35.2)**	**44 (34.4)**	**48 (37.5)**	**70 (54.7)**
Cis	**180 (19.5)**	**401 (43.4)**	**70 (7.6)**	**162 (17.6)**	**140 (15.2)**	**387 (41.9)**
Chi-square	13.355	7.447	87.697	20.189	38.168	7.446
*p*	**< 0.001**	**0.006**	**< 0.001**	**< 0.001**	**< 0.001**	**0.006**
*Cramer's V*	0.113	0.084	0.289	0.139	0.191	0.084
**Gender identity group**						
Trans women	**15 (45.5)**	20 (60.6)	**9 (27.3)**	**12 (36.4)**	**16 (48.5)**	**23 (69.7)**
Trans men	**19 (32.8)**	28 (48.3)	**20 (34.5)**	16 (27.6)	**22 (37.9)**	23 (39.7)
Trans non-binary	9 (24.3)	**24 (64.9)**	**16 (43.2)**	**16 (43.2)**	10 (27)	**24 (64.9)**
Cis women	**51 (13.6)**	**107 (28.5)**	**22 (5.9)**	**47 (12.5)**	**51 (13.6)**	**129 (34.4)**
Cis men	**125 (23.8)**	**286 (54.5)**	**44 (8.4)**	110 (21.0)	84 (44.7)	**248 (47.2)**
Cis non-conforming	4 (17.4)	8 (34.8)	4 (17.4)	5 (21.7)	5 (21.7)	10 (43.5)
Chi-square	31.761	70.498	96.059	33.917	45.201	32.058
*p*	**< 0.001**	**< 0.001**	**< 0.001**	**< 0.001**	**< 0.001**	**< 0.001**
*Cramer's V*	0.174	0.259	0.302	0.180	0.207	0.175
**Sexual orientation group**						
Heterosexual trans	**9 (39.1)**	14 (60.9)	**9 (39.1)**	8 (34.8)	**10 (43.5)**	11 (47.8)
Lesbian trans	**8 (53.3)**	8 (53.3)	**5 (33.3)**	**6 (40.0)**	**10 (66.7)**	10 (66.7)
Gay trans	5 (38.5)	9 (69.2)	**5 (38.5)**	5 (38.5)	3 (23.1)	**10 (76.9)**
Bisexual trans	10 (26.3)	20 (52.6)	**12 (31.6)**	11 (28.9)	**13 (34.2)**	17 (44.7)
Other trans	11 (28.2)	21 (53.8)	**14 (35.9)**	**14 (35.9)**	**12 (30.8)**	22 (56.4)
Lesbian cis	**34 (15.7)**	**68 (31.5)**	**14 (6.5)**	**30 (13.9)**	30 (13.9)	84 (38.9)
Gay cis	109 (23.0)	**254 (53.7)**	**38 (8.0)**	98 (20.7)	73 (15.4)	**224 (47.4)**
Bisexual cis	**29 (15.8)**	**62 (33.7)**	**11 (6.0)**	**25 (13.6)**	30 (16.3)	**64 (34.8)**
Other cis	8 (16.0)	17 (34.0)	7 (14.0)	9 (18.0)	7 (14.0)	**15 (30.0)**
Chi-square	26.625	50.330	91.755	28.090	51.211	26.159
*p*	**0.001**	**< 0.001**	**< 0.001**	**< 0.001**	**< 0.001**	**0.001**
*Cramer's V*	0.159	0.219	0.295	0.163	0.221	0.158

Significant differences are highlighted in bold.

As indicated in Table 2, trans persons suffered significantly more harassment than cis persons in all contexts, the health and family contexts having the highest differences (27.6 and 22.3 percentage points, respectively).

The disparity of harassment contexts according to gender identity and sexual orientation can also be seen in Table 2, where significant differences were found in all harassment contexts between the groups. In the analyses by gender identity, trans persons, especially trans women and trans non-binary, reported more harassment experiences than cis LGB persons in all contexts although with variations. Trans women and trans men showed significantly higher rates of harassment in the work setting than cis women and cis men. In the educational context, trans non-binary persons and cis men, both with rates above 50%, differed significantly from the harassment suffered by cis women, around 28%. In the health context, all the groups showed significant differences, except cis non-conforming, transgender groups being most affected by harassment experiences (27–43%) in comparison with cis women and cis men (6–8%). In the sports context, trans non-binary (43.2%) and trans women (36.4%) reported more harassment than cis women (13%). In the family setting, trans women and trans men showed higher percentages of harassment than cis women, who experienced least. Trans women and trans non-binary suffered more than twice as much as cis men and women in public places.

Taking into account the analyses by sexual orientation, except in the educational and the family settings, significant differences always occurred between heterosexual/LGB/other sexual orientation trans and LGB/other sexual orientation cis. These differences always showed higher percentages of harassment in trans people. In the educational context differences were found only between the LGB cis groups, with gays suffering more harassment than lesbians and bisexuals. In the family setting, differences were found only in the trans groups, with lesbian trans standing out in terms of harassment suffered in comparison with heterosexual trans, bisexual trans and other trans persons.

### Harassment risk profile

Binomial logistic regression analysis was used to define the probabilities of LGBT people experiencing harassment in comparison with the main groups of this community (lesbian cis, gay cis, bisexual cis and transgender), according to age and contexts (Table 3). Firstly, chronological age emerged as a harassment prediction variable, with differences between the contexts. In education and public spaces, harassment diminished between 2 and 3% each year, although in the workplace the risk of harassment increased 1.07 times as they got older.

**Table 3:** Prediction model of harassment experience by age and LGBT groups (*n* = 989).

	**Some kind**	**Workplace**	**Educational**	**Health**	**Sports context**	**Family**	**Public spaces**
	**OR (95% CI)**	**OR (95% CI)**	**OR (95% CI)**	**OR (95% CI)**	**OR (95% CI)**	**OR (95% CI)**	**OR (95% CI)**
Age	**0.97 (0.96–0.99)**	**1.02 (1.01–1.04)**	**0.97 (0.96–0.99)**	1.00 (0.98**–**1.03)	1.00 (0.98**–**1.01)	0.98(0.97**–**1.00)	**0.98 (0.97–0.99)**
LGBT group							
Trans women	**4.59 (1.93–10.92)**	**3.76 (1.65–8.55)**	**3.96 (1.79–8.75)**	**5.70 (2.11–15.39)**	**4.09 (1.75–9.52)**	**5.04 (2.24–11.33)**	**4.79 (2.11–10.86)**
Trans men	**2.14 (1.16–3.94)**	**2.78 (1.39–5.53)**	**1.90 (1.04–3.48)**	**8.12 (3.59–18.36)**	**2.61 (1.26–5.39)**	**3.22 (1.66–6.25)**	1.26 (0.68**–**2.33)
Trans NB	**2.90 (1.36–6.17)**	1.69 (0.71**–**4.02)	**4.03 (1.90–8.51)**	**11.50 (4.70–28.10)**	**5.21 (2.37–11.43)**	2.01 (0.87**–**4.62)	**3.75 (1.78–7.91)**
Lesbian cis	1.41 (0.93**–**2.13)	0.87 (0.49**–**1.54)	1.11 (0.72**–**1.72)	**1**.02 (0.44**–**2.34)	1.12 (0.62**–**2.03)	0.90 (0.51**–**1.60)	1.43 (0.93**–**2.18)
Gay cis	**2.42 (1.67–3.52)**	1.28 (0.79**–**2.09)	**2.94 (2.00–4.32)**	1.17 (0.57**–**2.43)	**1.74 (1.04–2.92)**	1.05 (0.64**–**1.73**)**	**2.04 (1.40–2.99)**
Bisexual cis	Ref.	Ref.	Ref.	Ref.	Ref.	Ref.	Ref.

OR, odds ratio; CI, confidence interval; Variable/Categories where *p* < 0.05 are marked in bold lettering.

Some kind of harassment: *R*^2^ = 0.02 (Hosmer and Lemeshow), 0.03 (Cox & Snell), 0.05 (Nagelkerke). −2Log = 1,320.06. Model $\chi_{\left(6\right)}^{2}$ = 39.816, *p* < 0.001.

Workplace: *R*^2^ = 0.03 (Hosmer and Lemeshow), 0.03 (Cox & Snell), 0.05 (Nagelkerke). −2Log = 1,359.87. Model $\chi_{\left(6\right)}^{2}$ = 35.382, *p* < 0.001.

Educational: *R*^2^ = 0.09 (Hosmer and Lemeshow), 0.06 (Cox & Snell), 0.08 (Nagelkerke). −2Log = 1,301.96. Model $\chi_{\left(6\right)}^{2}$ = 61.762, *p* < 0.001.

Health: *R*^2^ = 0.10 (Hosmer and Lemeshow), 0.06 (Cox & Snell), 0.14 (Nagelkerke). −2Log = 602.71. Model $\chi_{\left(6\right)}^{2}$ = 70.948, *p* < 0.001.

Sports context: *R*^2^ = 0.02 (Hosmer and Lemeshow), 0.02 (Cox & Snell), 0.04 (Nagelkerke). −2Log = 947.37. Model $\chi_{\left(6\right)}^{2}$ = 28.965, *p* < 0.001.

Family: *R*^2^ = 0.03 (Hosmer and Lemeshow), 0.03 (Cox & Snell), 0.06 (Nagelkerke). −2Log = 889.44. Model $\chi_{\left(6\right)}^{2}$ = 36.817, *p* < 0.001.

Public spaces: *R*^2^ = 0.02 (Hosmer and Lemeshow), 0.03 (Cox & Snell), 0.04 (Nagelkerke). −2Log = 1,324.40. Model $\chi_{\left(6\right)}^{2}$ = 33.240, *p* < 0.001.

Secondly, trans women (OR = 4.59), trans non-binary (OR = 2.90), lesbian cis (OR = 2.42) and trans men (OR = 2.14) were more likely to suffer harassment at some time in their lives than bisexual cis persons. The likelihood of suffering harassment varied in different contexts. Trans women (OR = 3.76) and trans men (OR = 2.78) were the only groups who were more likely to be harassed in the workplace than bisexual cis persons. In education, the highest risk of harassment was assigned to trans non-binary, closely followed by trans women, gay cis and trans men in comparison with bisexual cis persons. In the health context, trans non-binary were much more likely (11.5 times more) to suffer harassment, followed by the other trans groups (8.1 times more for trans men and 5.7 times more for trans women) taking bisexual cis persons as the reference group. The trans non-binary group again had the highest probability of suffering harassment (OR = 5.21) in the sports context, followed by trans women (OR = 4.09) and gays (OR = 1.74). In the family environment, only trans women (5.04 times more) and trans men (3.22 times more) showed more likelihood of suffering harassment than bisexual cis persons, while trans women, trans non-binary and gay cis were more likely (2–4.8 times more) to suffer harassment in public spaces than bisexual cis persons.

## Discussion

To our knowledge, this is the first study to empirically address the issue of harassment disparities in the adult Spanish LGBT population, and one of the first to analyze them comparatively according to age, gender identity, sexual orientation and perpetration contexts within the whole LGBT community, with special emphasis on the transgender collective.

### Overall prevalence of harassment

The overall prevalence of harassment shows that 54.4% of adult LGBT participants experienced harassment at some time in their lives. This percentage is midway between the 25.1% of non-heterosexual adolescents (55) and 59.9% of trans adults in Spain (24). This behavior is less prevalent in Spain than in Australia, which registers 85% homophobic harassment and violent conduct (17). However, the percentage in this study is higher than those indicated for the UK and USA (36 and 40%, respectively), although the aforementioned figures are for the previous 12 months (10, 59). It is remarkable that the prevalence in the present study is similar to the 57% of harassment registered for the previous 5 years among Spanish and 58% among the European LGBT communities (14, 15). This similarity is in line with the legal and policy situation of Spain in comparison with the whole Europe since this country is among the first quartile of European countries in the development of LGBT community rights, though decreasing in recent years (60). A key example that explain this similarity is the same sex marriage legal recognition that presented a 66.2% of support from Spanish population in 2004 (61).

However, the prevalence of harassment among Spanish LGBT participants is substantially higher than the total of hate crimes committed in this country (41.65%) during the last 5 years and include several motives, such as sexual orientation, gender identity, ideology and xenophobia, among others (62). This comparison suggests that the overall harassment in Spanish society is still far from free of homo and trans-negativity attitudes and behavior and it is assumed that more LGBT persons will suffer harassment than hetero-cis people, as reported in studies from other countries (25, 28, 37).

### Harassment by age, gender identity, and sexual orientation

More young adults (aged 25–34 years) are victims of harassment (61.3%) than in the older group (>50 years) (44.2%). This result is in line with other European studies that found that young people under 18 years suffer 9% more harassment than that of the entire LGBT community (14). Another USA study also found a higher rate of discrimination and harassment among the younger generation, ranging from 20% in Baby Boomers (between 58 and 76 years old) to 57% in Generation Z (under 26 years old) (10). A similar study on a Spanish sample of trans persons reached a similar conclusion since the participants who disclose their gender identities at a younger age experience higher percentages and frequency of harassment than those who disclose it at an older age (24). The period in which people disclose their gender identities and sexual orientations (“outness”) seems to be relevant in understanding disparities of age because youths and young adults are more comfortable in being out in public locations and thus experience a higher rate of harassment than other age groups (23).

Harassment disparities are also found between transgender (67.2%) and cisgender (LGB) (52.7%) participants, as are also observed in previous research. For instance, harassment and discrimination in transgender European people is more prevalent (48%) than in the European LGBTI members of the whole community (38%) (14). This difference in the prevalence of harassment and discrimination is also similar between transgender people in the US (62%) and the entire US LGBT community (36%) (10). Again, similar disparities are found among North-American transgender adults (90%) than in their non-transgender siblings, sisters (80%) and brothers (63%) (26). However, in the Spanish cultural context, the disparity observed between transgender and cisgender participants is probably due to the anti-trans rhetoric emerged during the debate of gender recognition law still not approved in the Parliament (63) compared with the increasingly accepted support toward LG collectives since the same sex legal recognition. Even so, transgender women emerge in the present study as the group with the highest percentage of harassment (75.8%), which significantly differs from that of cisgender women, the group with the lowest percentage (42.4%). This is an important result since it shows how trans women in Spain are doubly harassed and discriminated against for being trans and women or, as Serano (64) has pointed out, they suffer from misogyny for being women and rejection for not being cisgender men.

The differences in harassment between the nine sexual orientation subgroups are especially found between lesbian trans (80%) and the other LGB cis people (59.8% gay, 47.7% lesbian, 44% bisexual, and 38% other). These results are close to those found by Michel et al. (29) among US LGBT adolescents since lesbian/queer girls suffer a higher percentage of harassment (72%) than bisexual girls (66%) and gay/queer boys (66%). This is probably due to the role transgender lesbians play in breaking traditional gender and sexual norms in Western heteronormative societies more widely and Spain in particular (65).

### Harassment by context

The results of the present study show that educational and public spaces are the contexts in which LGBT people experience most harassment, while the least is experienced in family and health contexts. The highest prevalence is consistent with the European FRA (14) report, but only partially consistent with US studies that indicate public spaces and the workplace (10) or streets and home (23) as the contexts with the highest percentage. The lowest prevalence in the present study has no LGBT data to compare but is different to the prevalence in US and Spanish transgender people, who show the family and workplace as the contexts with the least harassment in the former country and sports and the workplace in the latter (24, 37). The results also show that more than half the participants experience harassment in more than one context (from two to four contexts: 50.2%), a higher percentage than that of trans persons (20.8%), who participated in a previous Spanish study (24). This result suggests that homo and trans-negativity environments are diffused through different primary and secondary socialization contexts in Spain and has probably increased in recent years, as corresponds in the decrease of the Rainbow Index on LGBT rights (60).

Significant harassment disparities based on gender identity were found in every context under study. Trans persons suffered significantly more harassment than cis LGB persons (men and women or women alone) in all types of contexts. Only cis gays experienced more harassment than cis lesbian and bisexual women in educational settings. Apart from the previous exception, the results of the present study are in line with the literature that found that trans men, trans women, non-binary or the combination of the three trans groups experience more harassment than cis LGB in general or in individual contexts (10, 11, 14, 26, 38, 39, 49, 66). In fact, these results reinforce the vulnerability of trans persons, compared with the cisgender groups of the LGBT community, observed in previous discussion section that suggest a more transphobic than homophobic environment in the recent years within Spanish society.

Significant harassment disparities by sexual orientation were also found in four of the six contexts studied between trans people with different orientations, especially lesbian and gay trans (not heterosexuals), compared to LGB/other cis orientation. This is probably due to the increased social acceptance and perceived equality achieved in Spain by homosexuals in recent decades (67). However, in the educational and family contexts, significant differences only appear among particular sexual orientation groups, i.e., in educational contexts differences were found between cis sexual orientation groups (gay cis the group with highest percentage of harassment, followed by bisexual and lesbian cis) and in family contexts the differences are mainly reported among trans groups (lesbian trans have the highest percentage of harassment, followed by bisexual cis and trans with other sexual orientations). In general, these results again show that disparities are mainly based on the gender identity of the participants, which indicates the highly discriminatory harassment suffered by trans people among the whole LGBT community. However, in the study by Whitfield et al. (23), bisexuals are identified as the group with highest rate of harassment in educational contexts and gays in street or public contexts, although this study does not include trans participants in its sample to know potential cross-cultural comparisons between the US and Spain in this issue.

### Risk profile of harassment

Chronological age emerged in the present study as a predicting variable of harassment in Spanish LGBT people, although it varies according to contexts. In education and public spaces, harassment is likely to diminish by between 2 and 3% as they get older, as in the case of Whitfield et al. (23), who found that harassment also decreases with age in both contexts in the US. On the other hand, the harassment in this study is likely to increase 1.07 times in the workplace as LGBT employees get older, probably due to less tolerance toward the old LGBT workforce compared with the young one in Western cultures.

Trans women, trans non-binary, lesbian cis and trans men were more likely to suffer harassment than bisexual cis persons. With the exception of lesbian cis, these results are consistent with the research that reports trans people of any group (trans men, trans women, trans non-binary) experience more harassment than their cis LGB peers (10, 14, 26). The likelihood of suffering harassment varies according to the context. Trans women present the highest risk of harassment in three contexts (workplace, family and public spaces) and trans non-binary in the other three contexts (educational, health and sport) compared with the bisexual cis that make up the reference group. This is probably due to the increased social acceptance of LGB people, since homonormativity is becoming part of cis-normativity in Western societies. While trans people still experience many types of discrimination and inequalities, lesbian, gay and bisexual cis are more likely to be accepted (68–70). This is also probably due to the less social contact cisgender people have with trans people compared to bisexual cis people, which can influence negative attitudes toward trans people and transphobic attitudes and increase the likelihood trans people suffer from harassment experiences (71).

## Limitations

In order to accurately interpret the results of this study, some potential weaknesses need to be considered. Firstly, it was not possible to form a representative sample because a reliable estimation of the number of LGBT people in Spain does not exist. However, we used the largest adult sample in a study from the specialized Spanish literature. Secondly, harassment was measured by a survey based on the perception of the participants instead of an objective measure based on the researchers' observations. Nevertheless, in the case of LGBT participants, this limitation is not an overrepresentation, as often happens in pattern studies. In fact, there is a risk of underrepresentation with this population since LGBT people are more likely to perceive harassment as normal in some situations (72). Thirdly, our participants were asked whether they had ever experienced harassment and not whether they had experienced it for a certain period of time (e.g., 5 years, last year, last month), as in other studies (10, 14, 59). Fourthly, harassment on social networks is not included among the contexts analyzed, when it is in fact emerging as an important harassment context. However, this new context can be included in the public space, which is part of the present study, although future research will require a virtual public space, mainly focused on adolescents or school contexts (27, 39, 55). Despite these limitations, the present study reveals new evidence and offers useful insights into the harassment experienced by LGBT people and the harassment risk profile in Spain, as well as extending the debate on harassment disparities to the international level.

## Conclusions and implications

The results of this study reveal that harassment is a serious problem for LGBT adults in Spain, since 54.4% of the sample experienced harassment at some time in their lives. Significant harassment disparities were found by age, sexual identity and sexual orientation, with more young adults experiencing harassment than the older group, and more transgender than cisgender people in the LGBT community. According to the particular gender groups in the sample, transgender women emerged as the group with the highest rate of harassment and cisgender women the group with the lowest. The differences in harassment between the nine sexual orientation groups were especially found between lesbian trans and the other LGB cis people. Educational premises and public spaces were the contexts with the highest percentage of harassment, while the family and health systems had the lowest percentage. More than half the participants experienced harassment in more than one context. More trans persons suffered significant harassment than cis LGB persons in all the types of contexts studied. Significant disparities were found among sexual orientation in four of the six contexts analyzed between trans people with different orientations (especially lesbian and gay trans) compared with LGB cis. Chronological age was found to be a predicting variable of harassment. In education and public spaces harassment is likely to diminish between 2 and 3% each year as age increases, but is 1.07 times higher in the workplace as LGBT employees age. Trans women, trans non-binary, lesbian cis and trans men were more likely to suffer harassment than bisexual cis persons. By contexts, trans women present the highest risk of harassment in the workplace, family and public spaces and trans non-binary in the educational, health and sports compared to the bisexuals in the group of reference.

The results reveal that harassment is a serious problem for LGBT adults in Spain. Trans people suffer the highest percentage of harassment in the LGBT community, especially the younger group and in all the contexts analyzed. These data suggest the existence of a more transphobic than homophobic situation in Spain, an important concern for public policies. From the health disparities perspective, this means that trans people's characteristics and attributes differ from those of the sexual minorities mainstream. Spanish programs and policies targeted for improving health should thus consider the differences found in this study. In fact, these results on harassment disparities can be useful in evaluating disadvantages in health minorities such as the LGBT community, and especially the differences that affect trans people. Although evaluating harassment disparities is not a customary practice in research, in Spain and elsewhere, it could be useful to guide decision-making at the local and national level, as well as to justify the investments required based on the evidence.

## Data availability statement

The original contributions presented in the study are included in the article, further inquiries can be addressed to the corresponding author.

## Ethics statement

The studies involving human participants were reviewed and approved by Ethics Committee of the University of Valencia and the Catalan Sports Council. The participants provided their written informed consent to participate in this study.

## Author contributions

JD-D, SP-G, and JG-Q were involved in setting up this study and elaborating theoretical framework. JD-D and AV-P defined general methodological issues. AV-P contributed especially to the data analysis and results section. JG-Q was involved in data collection. AV was in charge of project administration. All authors contributed to the revision of the article and approved the submitted version.
